# Ductility Improvement of an AZ61 Magnesium Alloy through Two-Pass Submerged Friction Stir Processing

**DOI:** 10.3390/ma10030253

**Published:** 2017-03-02

**Authors:** Xicai Luo, Genghua Cao, Wen Zhang, Cheng Qiu, Datong Zhang

**Affiliations:** 1 National Engineering Research Center of Near-net shape Forming for Metallic Materials, South China University of Technology, Guangzhou 510640, China; meluoxicai@mail.scut.edu.cn (X.L.); jack_eei@scut.edu.cn (W.Z.); cqiu@scut.edu.cn (C.Q.); 2 Guangdong Key Laboratory for Advanced Metallic Materials Processing, South China University of Technology, Guangzhou 510640, China; cghcaogenghua@126.com

**Keywords:** AZ61 magnesium alloys, friction stir processing, microstructure, texture, mechanical property

## Abstract

Friction stir processing (FSP) has been considered as a novel technique to refine the grain size and homogenize the microstructure of metallic materials. In this study, two-pass FSP was conducted under water to enhance the cooling rate during processing, and an AZ61 magnesium alloy with fine-grained and homogeneous microstructure was prepared through this method. Compared to the as-cast material, one-pass FSP resulted in grain refinement and the β-Mg_17_Al_12_ phase was broken into small particles. Using a smaller stirring tool and an overlapping ratio of 100%, a finer and more uniform microstructure with an average grain size of 4.6 μm was obtained through two-pass FSP. The two-pass FSP resulted in a significant improvement in elongation of 37.2% ± 4.3%, but a slight decrease in strength compared with one-pass FSP alloy. Besides the microstructure refinement, the texture evolution in the stir zone is also considered responsible for the ductility improvement.

## 1. Introduction

Improving the ductility of magnesium alloys through grain refining has drawn great interest, as the application of magnesium alloys is generally limited by their poor formability. In 1999, Mishra et al. [1] firstly proposed that friction stir processing (FSP) could be used as a new technique for grain refinement. Since then, fine-grained metallic materials including Al, Mg and Ti alloys prepared by FSP have been studied extensively, and the properties of these materials are generally improved due to microstructure refinement [2,3,4]. FSP is an effective and efficient method of preparing fine-grained magnesium alloys, according to the literatures [1,5,6]. Based on FSP, some modified methods have been developed to further decrease the grain size by: (1) enhancing the cooling rate during FSP through copper backing plate with higher thermal conductivity, water or liquid nitrogen with higher heat absorbility [7,8,9]; and (2) conducting two or more FSP passes on base material (BM), i.e., multi-pass FSP (MFSP) [10,11,12]. Dadashpour et al. [10] investigated the effect of pass number on the microstructure and properties of FSP AZ91C Mg alloy and attributed the enhancement of mechanical properties to reinforcement of the second phase and homogenization of microstructure. Table 1 shows a summary of research on magnesium alloys prepared through MFSP. Besides grain refinement, MFSP can also be used to repair the defects that appear in the previous processing [13].

As a modified FSP technique, the effects of MFSP including cooling mediums [17], geometric profile of tool pin [18] and processing parameters [16] on the microstructure and mechanical behavior of magnesium alloys have been investigated. After the first pass of FSP, the refined microstructure has two opposite evolution tendencies in subsequent FSP: (1) further refinement through dynamic recrystallization (DRX) due to severe plastic deformation (SPD); and (2) grain coarsening due to the accumulative heat input. Sometimes, further grain refinement cannot be achieved by simply increasing passes. Dadashpour et al. [10] found that the grain size of MFSP AZ91C Mg alloy increased as the pass number increased, without any cooling medium. Therefore, in the design of MFSP, the grain coarsening effects need to be considered due to the heat input of the subsequent pass. Bhargava et al. [16] illuminated the effect of the first pass and second pass with different processing parameters on the texture variation and tensile strength of a rolled AZ31 alloy. Du et al. [9] applied two-pass FSP with rapid cooling medium (liquid nitrogen), and obtained an average grain size of 100 nm in an AZ61 magnesium alloy.

Submerged FSP (SFSP) is conducted under water, and the processing temperature is lower than that of normal FSP (NFSP) [19]. Hofmann et al. [8] prepared an Al-6061-T6 alloy with a grain size less than 200 nm by SFSP and supposed that SFSP could refine grains and improve the mechanical properties. Chai et al. [20] produced an AZ91 alloy with an average grain size of 1.2 µm through SFSP, while the average grain size of NFSP sample was ~7.8 µm. During submerged friction stir processing, the shoulder of the tool makes firm contact with the materials after the pin inserted into the plates, and the process is finished in a few minutes. Therefore, the possibility of corrosion caused by water is limited. In addition, Chai et al. [20] reported that the surfaces of the SFSP AZ91 alloy were relatively clean and no perceivable corrosion was mentioned in their paper. According to the anodic polarization curves in dilute electrolyte (0.001 N NaCl solution), the corrosion susceptibility of as-cast AZ61 magnesium alloy is similar to the as-cast AZ91 alloy [21], so SFSP can also be applied to the as-cast AZ61 magnesium alloy for microstructure refinement. It is considered that finer grains may be obtained by repeating SFSP, i.e., multi-pass SFSP. However, research on multi-pass SFSP has been rarely reported to present. In this study, two-pass SFSP was conducted on cast AZ61 magnesium alloy with a smaller tool for the second pass (a larger tool used in the first pass), to reduce the heat input during the second pass FSP. Microstructure and tensile behavior of the two-pass SFSP AZ61 alloy were investigated.

## 2. Results

### 2.1. Microstructure Observation

**Figure 1:**
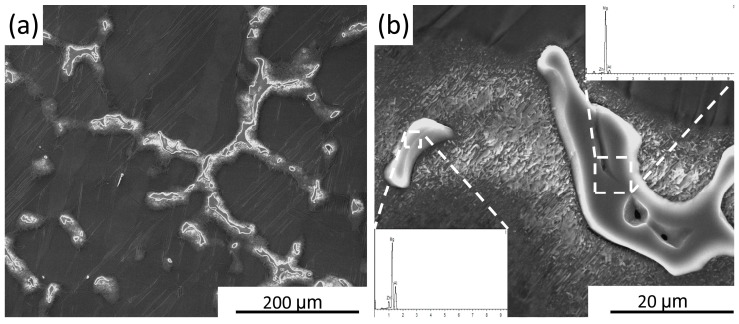
Scanning electron microscopy (SEM) images of the as-cast AZ61 specimen at (**a**) low magnification and (**b**) high magnification

**Figure 2:**
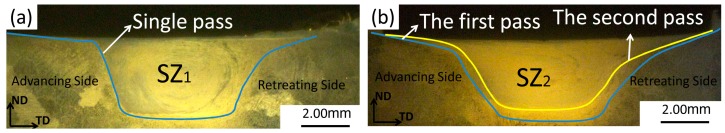
Macrographs of as-FSP specimens: (**a**) one-pass SFSP; (**b**) two-pass SFSP.

Microstructure of the as-cast AZ61 alloy is composed of α-Mg grains and coarse β-Mg_17_Al_12_ phase distributed at the grain boundaries, as shown in Figure 1. Figure 1b presents the morphological characteristics of second phase, and their composition is identified by energy-dispersive spectroscopy (EDS). Figure 2 presents the cross-sectional macrographs of one-pass and two-pass SFSP specimens, where the processing profiles of the respective pass can be seen clearly. No defect is found in the samples. Although magnesium alloys are susceptible to corrosion when in contact with water, no evidence of corrosion was found in our processed samples. In the stirred zone (SZ) of the one-pass SFSP specimen, the onion ring pattern can be seen clearly, which is similar to the material flow trace during SFSP, as shown in Figure 2a. Since the second pass was conducted with a smaller pin, the processing region (SZ_2_) is totally inside SZ_1_ (Figure 2b). This shows that the material in SZ_2_ is relatively homogenous after the second-pass SFSP.

**Figure 3:**
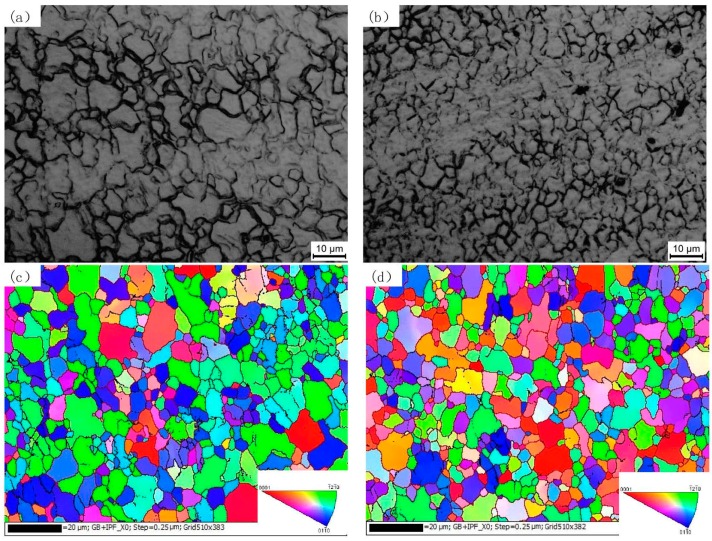
Microstructures in stirred zones (SZs) of SFSP AZ61 alloy specimens examined by optical microscopy (OM) and electron backscattered diffraction (EBSD): (**a**,**c**) for one-pass; (**b**,**d**) for two-pass specimens.

Figure 3 shows the microstructures in the SZ of the FSP AZ61 alloy examined by optical microscopy (OM) and electron backscattered diffraction (EBSD). Compared with the as-cast microstructure (Figure 1a), α-Mg grains are greatly refined after SFSP. The average grain size of one-pass and two-pass SFSP alloys are 5.2 μm and 4.6 μm, respectively. That is to say, further grain refinement is achieved by two-pass SFSP to some extent.

**Figure 4:**
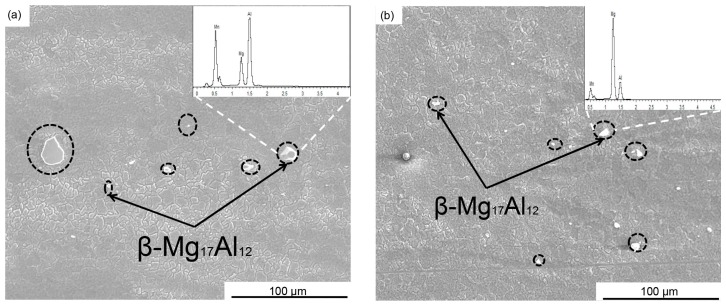
SEM micrographs in SZ of AZ61 Mg alloy samples: (**a**) one-pass SFSP and (**b**) two-pass SFSP specimens.

**Figure 5:**
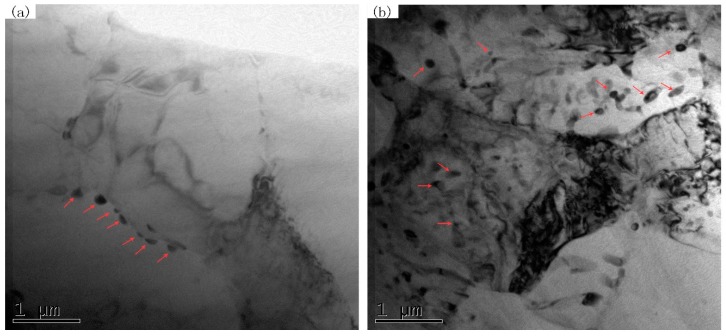
Transmission electron microscopy (TEM) images showing particles as marked by arrows in SZ of (**a**) one-pass and (**b**) two-pass SFSP specimens.

Figure 4 shows the morphological characteristics of the second phase within SZ after SFSP. From Figure 4a, the second-phase particles with different sizes can been found in the SZ of one-pass SFSP sample, which are formed through the breakup of coarse phases during SFSP. After two-pass SFSP, the remaining large particles are further refined, as shown in Figure 4b. From the transmission electron microscopy (TEM) image shown in Figure 5, fine second-phase particles can be seen in the two SFSP samples. In the one-pass SFSP sample, some small particles are located at the grain boundaries (Figure 5a) and after two-pass SFSP fine second-phase particles are found in the interior of grains as shown in Figure 5b.

### 2.2. Texture Analysis

**Figure 6:**
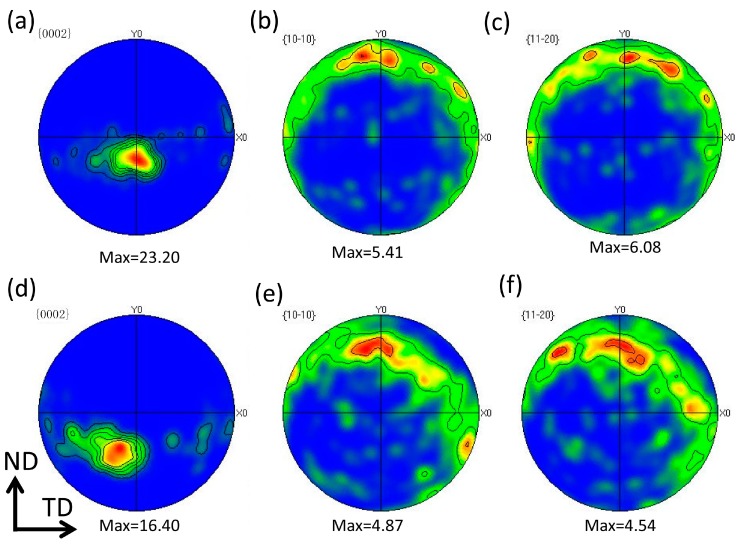
{0002}, {10-10} and {11-20} pole figures of a (**a**–**c**) one-pass and (**d**–**f**) two-pass FSP AZ61 alloys. ND: normal direction; TD: transverse direction.

Figure 6 displays the {0002}, {10-10} and {11-20} pole figures on the T-plane of one-pass and two-pass SFSP samples, respectively. Detailed statistical results of the texture and the angle between the *c*-axis with transverse direction (TD) or processing direction (PD) for the basal plane in FSP AZ61 samples are summarized in Table 2. The *c*-axis of grains in SZ of the one-pass SFSP specimen are perpendicular to TD and tilted to normal direction (ND) about 19° away from the PD. In contrast, the *c*-axis of grains in the two-pass SFSP specimen rotates ~35° away from PD to ND as well as ~14° away from PD to TD, as shown in Figure 6d. The {0002} pole figure of the one-pass SFSP alloy has a higher maximum intensity of 23.2 multiples of a random density (MRD) in comparison with 16.4 MRD in two-pass SFSP specimen. Compared to the {0002} basal plane, orientation distributions of {10-10} and {11-20} planes are not so obvious.

### 2.3. Mechanical Properties

**Figure 7:**
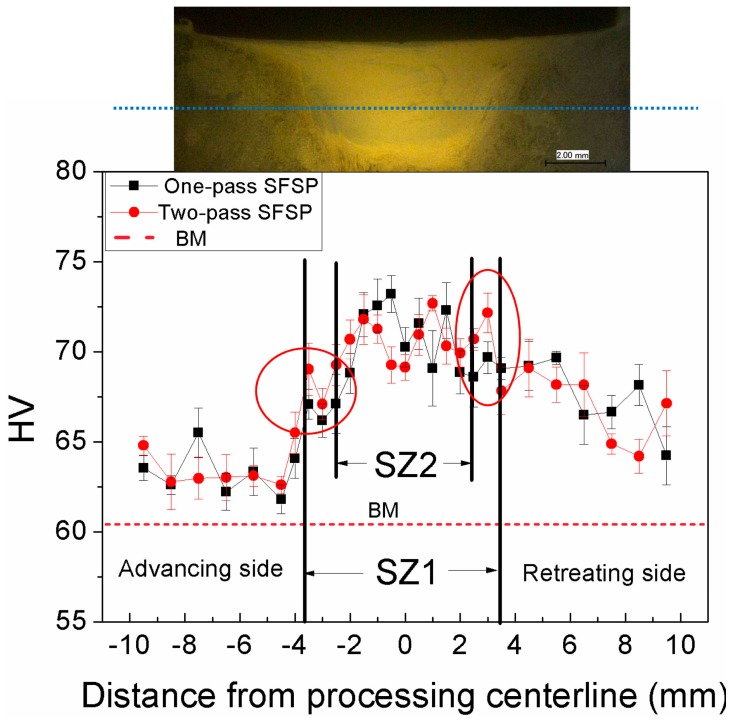
Vicker’s microhardness distribution of AZ61 alloy presents in different pass FSP conditions. BM: base material.

Figure 7 shows the Vicker’s microhardness distribution on the cross-section of experimental alloys. The average Vicker’s microhardness of BM is 61 HV due to its coarse dendritic structure, while the hardness of SZ in the one-pass and two-pass SFSP samples increases to 71 ± 0.4 HV and 70 ± 1.0 HV, respectively. Compared to BM, the hardness of the SFSP specimens is greater, which is mainly attributed to grain refinement.

**Figure 8:**
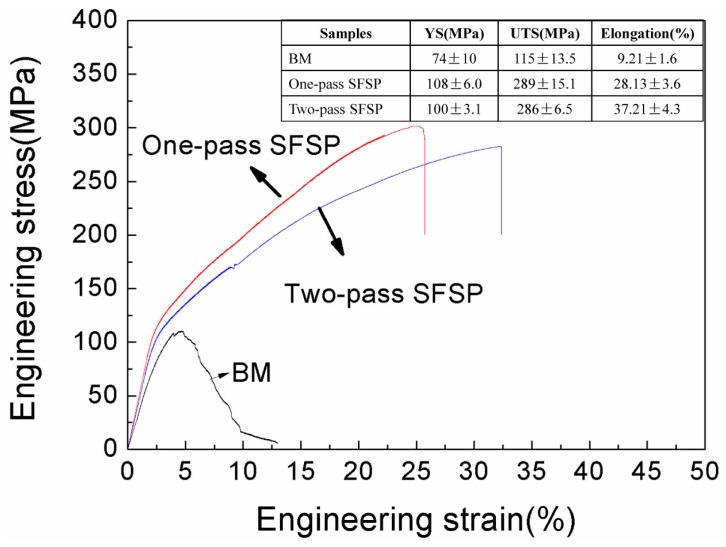
Tensile stress-strain curves for BM and the SFSP AZ61 specimens. YS, UTS and elongation to fracture of BM, one-pass, and two-pass SFSP specimens are listed in the table.

Figure 8 summarizes the room-temperature tensile properties of the BM, one-pass and two-pass SFSP specimens. The BM exhibits lowest mechanical properties in terms of yield strength (YS) of 74 ± 10 MPa, ultimate tensile strength (UTS) of 115 ± 13.5 MPa and elongation of 9.2% ± 1.6%, due to the coarse grains and large second phase networks. After one-pass and two-pass SFSP, the YS, UTS and elongation are improved to 108 ± 6.0 MPa, 289 ± 15.1 MPa, 28.1% ± 3.6% and 100 ± 3.1 MPa, 286 ± 6.5 MPa, 37.2% ± 4.3%, respectively. Compared to BM, the tensile properties of SFSP specimens are all improved significantly. Particularly, the ductility of the two-pass SFSP AZ61 alloy is relatively good, as compared to the MFSP magnesium alloys given in Table 1.

**Figure 9:**
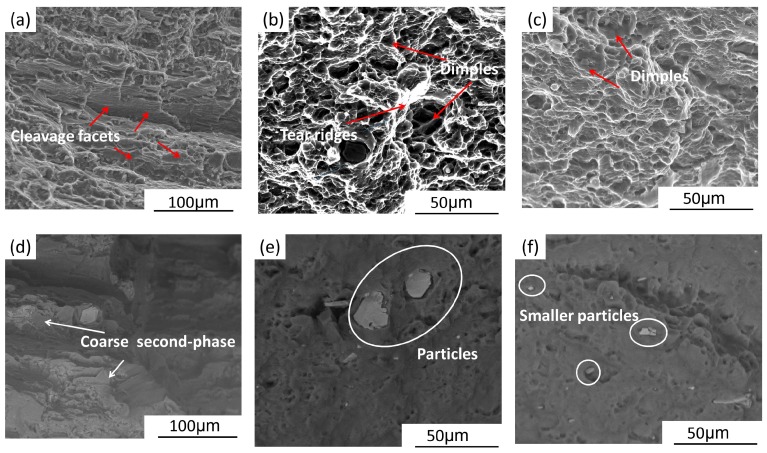
Surface characteristics of the fractured specimens after tensile tests of (**a**,**d**) BM; (**b**,**e**) one-pass SFSP; (**c**,**f**) two-pass SFSP specimens.

Figure 9 shows the tensile fracture morphologies of test specimens. Cleavage facets (as marked by arrows) can be seen clearly on the fractured surface of BM (Figure 9a), and the fracture originates from the coarse second phase, as shown in scanning electron microscopy (SEM) backscattered image (Figure 9d). The BM fails through brittle fracture mode judged from these typical characteristics. This is the main reason for the low ductility in BM. Figure 9b,e exhibits the fracture morphology of one-pass SFSP specimen. Note that the dimples and tearing ridges distribute dispersedly on the transverse section and some coarse second phases particles can be seen on the fracture surface. Both the one-pass and two-pass SFSP specimens failed through ductile fracture mode. However, there are more dimples, tearing ridges and smaller particles on the fracture surface of the two-pass SFSP specimen, as shown in Figure 9c,f. The fracture surface observation is in agreement with the results of the tensile test at room temperature.

## 3. Discussion

### 3.1. Effect of Multi-Pass Friction Stir Processing on Microstructural Evolution

**Figure 10:**
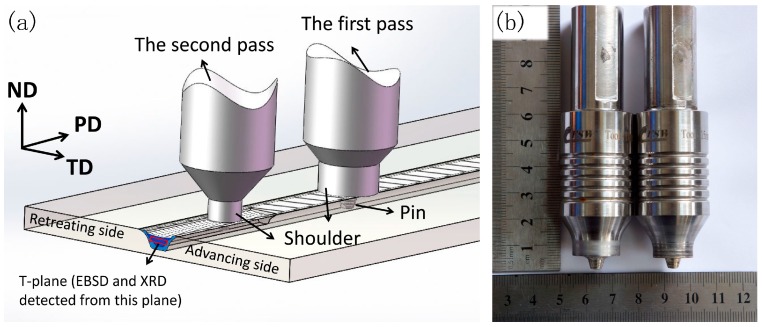
(**a**) Schematic illustration of two-pass SFSP and (**b**) the stirring tools used in the experiment.

It is well known that the intense plastic strain and heat input have significant effects on the microstructural evolution in the SZ during FSP. Many works have proven that FSP can effectively refine and modify the microstructure of casting magnesium alloys [22,23]. Sometimes, normal MFSP with constant processing parameters in subsequent passes cannot achieve grain refinement. This is attributed to the accumulated heat accompanying the multiple passes, leading to an increase in grain size [24]. An equation combining processing temperature and strain rate is expressed by the Zener–Hollomon parameter.

(1) $$Z=\dot{\varepsilon }\exp(Q/RT)$$

where $\dot{\varepsilon }$ is the strain rate, *R* the gas constant, *T* the temperature, and *Q* is the related activation energy. Chang et al. [25] concluded the grain size of FSP AZ31 alloy and *Z* parameter using the following equation:

ln *d* = 6.0 − 0.17 ln *Z* (2)

where *d* is the average grain size (in μm). According to Equations (1) and (2), grains could be refined by the process with an increase of *Z* parameter in terms of increasing $\dot{\varepsilon }$ or decreasing *T*. In order to achieve grain refinement, MFSP conducted on casting magnesium alloys needs to involve suitable processing parameters with higher *Z* values. In one hand, adopting lower rotation speed (ω) or higher traverse speed (υ) in subsequent passes generates lower heat input. Bhargava et al. [16] prepared a fine-grained microstructure with a lower ω/υ ratio in the second pass as compared with the first-pass FSP. On the other hand, it is a feasible way to conduct multi-pass FSP, with a smaller tool pin in the subsequent passes as compared to the first pass. Commin et al. [26] reported that using a tool with larger shoulder during FSP led to more heat input. Keeping the other parameters constant, smaller tool will produce less heat input and thereby decrease the processing temperature. In this work, two-pass SFSP used a smaller tool for the second pass, as the schematic illustration shows in Figure 10. Because of an increase in the *Z* parameter, two-pass SFSP achieved a finer grain size (4.6 μm) compared to one-pass SFSP alloy (5.2 μm).

Another function of MFSP is to homogenize the microstructure. On undergoing one-pass SFSP, β-Mg_17_Al_12_ phases in the BM were broken into small particles, while some large particles remained in SZ, as shown in Figure 4a. After two-pass SFSP, most of the large particles disappeared. The stirring effect in the subsequent pass is considered as the main reason for the particle refinement. Furthermore, dissolution and re-precipitation also play a role in microstructure evolution. It is reported that some β-Mg_17_Al_12_ in the SZ dissolved into α-Mg matrix during FSP due to the heat input [26]. Fine particles may precipitate from the supersaturated Mg matrix during FSP. From the TEM image shown in Figure 5b, it can be seen that fine particles with a size of about 140 nm exist in the interior of α-Mg grains, indicating reprecipitation took place in second-pass SFSP. The microstructure refinement and homogenization are beneficial to the mechanical properties of the AZ61 magnesium alloy.

### 3.2. The Relation of Microstructure, Texture and Mechanical Behavior

**Figure 11:**
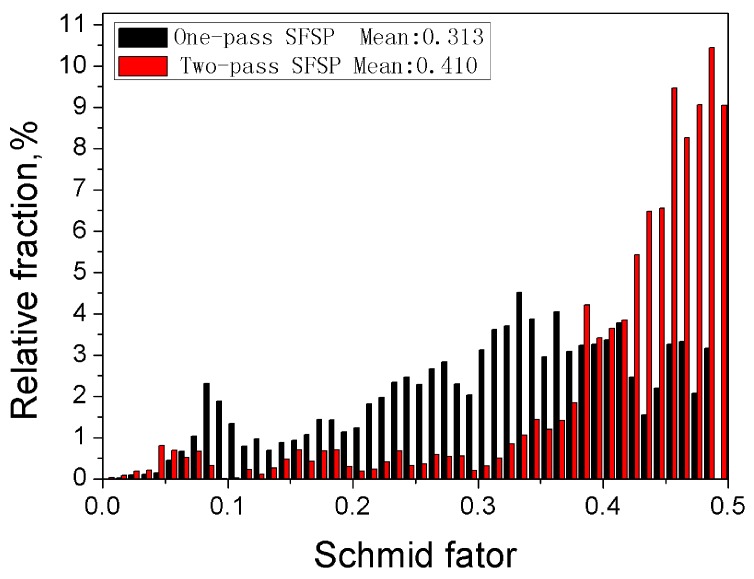
The relative fraction of any orientation factor accounts for all basal texture of SFSP samples.

It has been extensively reported that FSP can refine the microstructures of cast Mg alloys, and consequently improve their tensile properties according to Hall–Petch relationship. Therefore, it is easy to understand the strength and ductility improvements of one-pass and two-pass SFSP specimens as compared to the BM. It is worth noting that the average grain size of the two-pass SFSP specimen (~4.6 μm) is finer than that of the one-pass SFSP specimen (~5.2 μm), with their tensile strength decreased slightly, while the elongation of the two-pass SFSP specimen is 32.4% higher than that of that of the one-pass SFSP specimen. Wang et al. [27] reported weak grain size dependence of YS in the FSP AZ31 specimens as compared to the extruded specimens. When the grain size is refined to some extent, the effect of grain size difference on the strength of FSP specimen may be weakened. In order to understand the relationship between microstructure and mechanical behavior, texture should be taken into consideration. From the result listed in Table 2, the material in the SZ of the one-pass SFSP sample displays strong texture with the *c*-axis tilted towards PD by about 19°. In comparison, the *c*-axis of grain in SZ of two-pass sample exhibited a tilted angle of about 35° away from PD to ND. The preferred texture orientation of two-pass SFSP sample promotes basal slip easily, which affects plastic deformation behavior greatly. Therefore, the elongation of two-pass SFSP specimen obtained a great improvement. According to the texture characterization, tensile behavior is not only related to the slip system but is also associated with the Schmid factor [28]. The critical resolved shear stress is given by [29]:

τ = σ(cosφcosλ)_max_ (3)

where σ is the magnitude of the applied tensile stress, τ is resolved shear stress as a property of the material, and φ and λ are the angles between the stress axis and the slip direction and slip plane normal, respectively. The Schmid factor is defined as (cosφcosλ)_max_. Supposing the slip process takes place in system with (φ + λ) equal to 90° and there only exists one ideal basal texture over the entire sample, the Schmid factor can be calculated using a similar method reported by Mishra et al. [30]. In this work, the Schmid factor was calculated through software equipped in EBSD equipment, and the results are shown in Figure 11. The average Schmid factor for the basal plane slip system in the one-pass and two-pass SFSP samples are about 0.313 and 0.410, respectively. According to Equation (3), the strength in the two-pass SFSP sample is lower than that of the one-pass sample. The slip system with lower critical resolved shear stress and high Schmid factor usually starts first when plastic deformation takes place. The grain refinement effect may be weakened by the texture softening in the two-pass SFSP specimen. Therefore, its strength is a little lower than that of the one-pass SFSP specimen.

## 4. Materials and Methods

As-cast AZ61 magnesium alloy sheets of 6.5 mm in thickness were used in this study, and its chemical composition is Mg-6.80Al-0.79Zn-0.25Mn (wt. %). FSP was conducted on welding machine (FSW-3LM-003, FSW Technology Co. Ltd., Beijing, China) equipped with a cooling tank, in which the plate was completely submerged in room-temperature water. The flow speed of water was 40 ± 5 mL/s during the processing. FSP was carried out at a constant tool rotation speed of 800 revolutions per minute (rpm) and a tool traverse speed of 240 mm per minute with a 2.5° tool tilt for both passes. The stirring tool for the first pass had a shoulder of 18 mm in diameter, a threaded conical pin of 7 mm in root diameter and 5 mm in length. The second pass was conducted in the same way with 100 pct overlapping using a smaller tool, with a shoulder of 15 mm in diameter, a threaded conical pin of 6 mm in root diameter and 4 mm in length. The schematic graph of the two-pass SFSP is shown in Figure 10a and the stirring tools used in this study are shown in Figure 10b. 

Microstructures of FSP samples, with the cross section perpendicular to PD were examined by optical microscopy (OM, VHX-600, Keyence, Osaka, Japan), scanning electron microscopy (SEM, Nova Nano430, FEI, Hillsboro, OR, USA) equipped with energy-dispersive spectroscopy (EDS, Inca300, Oxford, UK) and transmission electron microscopy (TEM, JEM-2010, JEOL, Tokyo, Japan). The specimens for OM and SEM were etched in a solution of 8 mL ethanol, 10 mL distilled water, 10 mL acetic acid and 5 g picric acid. Thin TEM foils were prepared via an ion-miller (PIPS-691, Gatan, Pleasanton, CA, USA) at a voltage of 4 kV. Electron backscattered diffraction (EBSD) was used to examine crystallographic orientation distribution. Samples for EBSD were prepared by ion-etched method and the T-plane in SZ shown in Figure 10 was examined by SEM (S-3400N, Hitachi, Tokyo, Japan) operating at 20 kV. The resultant pole figures were determined through the HKL-Channel 5 software attached in the SEM. Coordinate axes of the pole figures are indicated using the PD, TD and ND of the sheet (Figure 10a). The average grain sizes of the specimens were measured by the mean linear intercept method and statistical analysis of EBSD results. Phase analysis of the specimens from the T-plane within the SZ was carried out by X-ray diffraction (XRD) (D8 ADVANCE, Bruker Corp., Billerica, MA, USA) with Cu K_α_ radiation.

**Figure 12:**
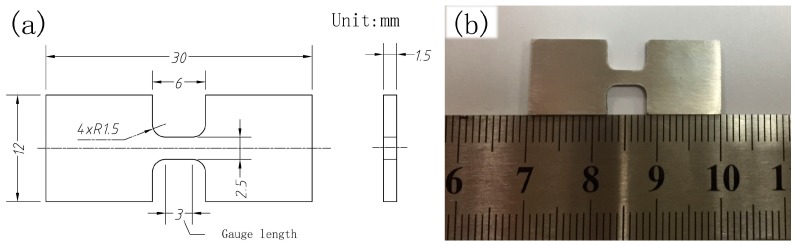
(**a**) Dimensions and (**b**) photograph of specimens for tensile test.

The Vicker’s microhardness tests were carried out along the central axis on the cross-section of the specimens. A load of 0.98 N with 10 s of loading cycle was adopted in the microhardness measurement. The indention interval was 0.5 mm in SZ and 1 mm in the other regions. Each indentation was measured three times and the average value was calculated as result. The dog-bone-shaped tensile specimens with a gauge dimension of 2.5 mm × 1.5 mm × 3 mm (width × thickness × length) were machined parallel to PD with the gauge completely within SZ_2_, as shown in Figure 12. Tensile tests were performed on a machine (AGS-X, Shimadzu, Kyoto, Japan) with a strain rate of 1.67 × 10^−3^ s^−1^. At least five specimens were tested to evaluate the average property values. Tensile fracture morphologies of failed specimens were observed by SEM as mentioned above.

## 5. Conclusions

Microstructure and mechanical properties of the AZ61 alloy prepared by one-pass and two-pass SFSP are investigated in the present work. The conclusions are summarized as follows:

1. One-pass SFSP resulted in grain refinement and breakup of β-Mg_17_Al_12_ phase. A finer and more uniform microstructure with an average grain size of 4.6 μm was obtained through two-pass SFSP.
2. Compared to as-cast AZ61 alloy, the mechanical properties of SFSP specimens were improved due to the grain refinement and precipitation strengthening. Furthermore, the elongation of two-pass SFSP specimen was remarkably increased to 37.2% ± 4.3% with a bit loss in strength as compared to the one-pass SFSP alloy.
3. Texture evolution during one-pass and two-pass SFSP caused the basal plane (0002) to be aligned with the angle ~19° and ~35° between *c*-axis of grains and PD, respectively. The orientation of basal plane in the SZ of the two-pass SFSP sample is aligned for easy slip, which leads to a higher ductility.

## Figures and Tables

**Table 1:** The summary of magnesium alloys prepared through multi-pass friction stir processing (MFSP), with an overlapping ratio of 100% with different cooling systems.

Material	Processing	Cooling	Grain Size (μm)	UTS ^1^ (MPa)	YS ^2^ (MPa)	Elongation (%)	Ref.
**AZ91**	Pre-heating + two-pass FSP	Copper plate + air	0.7	318	181	9.5	[7]
**AZ61**	Two-pass FSP	Liquid nitrogen	0.1	-	-	-	[9]
**Cast AZ80**	Two-pass FSP	Air	10.5	327.3	136.7	25	[11]
**Cast AZ61**	Four-pass FSP	Air	7.8	327	140	18	[14]
**Cast AZ91**	Two-pass FSP + aging	Air	15	337	177	10	[15]
**Rolled AZ31**	Two-pass FSP	Air	1.14	302	282	23.2	[16]

^1^ ultimate tensile strength; ^2^ yield strength. Ref.: reference.

**Table 2:** Detailed statistical results about the texture in SFSP alloys and the angle between the *c*-axis and TD or ND.

Surface	Samples	{0002}		{0002}	{10-10}	{11-20}
		Angle between *c*-axis ^1^ and TD/°	Angle between *c*-axis and PD/°	(Max Values of the Pole Figures in MRD)		
T-plane	One-pass SFSP	~90	~19	23.20	5.41	6.08
	Two-pass SFSP	~76	~35	16.40	4.87	4.54

^1^ parallel to normal direction of (0002) plane. MRD: multiples of a random density; PD: processing direction.
